# The Acceptability Among Health Researchers and Clinicians of Social Media to Translate Research Evidence to Clinical Practice: Mixed-Methods Survey and Interview Study

**DOI:** 10.2196/jmir.4347

**Published:** 2015-05-20

**Authors:** Jacqueline Tunnecliff, Dragan Ilic, Prue Morgan, Jennifer Keating, James E Gaida, Lynette Clearihan, Sivalal Sadasivan, David Davies, Shankar Ganesh, Patitapaban Mohanty, John Weiner, John Reynolds, Stephen Maloney

**Affiliations:** ^1^Monash UniversityFrankstonAustralia; ^2^Monash UniversityMelbourneAustralia; ^3^Canberra UniversityCanberraAustralia; ^4^Monash UniversitySunwayMalaysia; ^5^University of WarwickCoventryUnited Kingdom; ^6^SVNIRTAROdishaIndia

**Keywords:** social media, evidence-based medicine, communication, eLearning

## Abstract

**Background:**

Establishing and promoting connections between health researchers and health professional clinicians may help translate research evidence to clinical practice. Social media may have the capacity to enhance these connections.

**Objective:**

The aim of this study was to explore health researchers’ and clinicians’ current use of social media and their beliefs and attitudes towards the use of social media for communicating research evidence.

**Methods:**

This study used a mixed-methods approach to obtain qualitative and quantitative data. Participation was open to health researchers and clinicians. Data regarding demographic details, current use of social media, and beliefs and attitudes towards the use of social media for professional purposes were obtained through an anonymous Web-based survey. The survey was distributed via email to research centers, educational and clinical institutions, and health professional associations in Australia, India, and Malaysia. Consenting participants were stratified by country and role and selected at random for semistructured telephone interviews to explore themes arising from the survey.

**Results:**

A total of 856 participants completed the questionnaire with 125 participants declining to participate, resulting in a response rate of 87.3%. 69 interviews were conducted with participants from Australia, India, and Malaysia. Social media was used for recreation by 89.2% (749/840) of participants and for professional purposes by 80.0% (682/852) of participants. Significant associations were found between frequency of professional social media use and age, gender, country of residence, and graduate status. Over a quarter (26.9%, 229/852) of participants used social media for obtaining research evidence, and 15.0% (128/852) of participants used social media for disseminating research evidence. Most participants (95.9%, 810/845) felt there was a role for social media in disseminating or obtaining research evidence. Over half of the participants (449/842, 53.3%) felt they had a need for training in the use of social media for professional development. A key barrier to the professional use of social media was concerns regarding trustworthiness of information.

**Conclusions:**

A large majority of health researchers and clinicians use social media in recreational and professional contexts. Social media is less frequently used for communication of research evidence. Training in the use of social media for professional development and methods to improve the trustworthiness of information obtained via social media may enhance the utility of social media for communicating research evidence. Future studies should investigate the efficacy of social media in translating research evidence to clinical practice.

## Introduction

The importance of evidence-based practice (EBP) in health professions for providing patients with safe and effective care is well established [1]. Yet research that should change practice is often ignored, poorly implemented [1,2], or implemented only after significant time delay [3]. With an estimated 86% of relevant research evidence failing to be adopted into clinical practice [3], innovations to improve knowledge translation may assist in bridging the gap between health care knowledge and practice.

Barriers to the timely implementation of research into clinical practice include both a lack of awareness and acceptance of new research findings by those delivering patient care [1]. Establishing strong connections that enhance communication, collaboration, and education between health researchers, clinicians, health care organizations, educational institutions, and policy makers may foster practice that is grounded in evidence and ensure that ongoing research is relevant to clinicians. Social media may provide an avenue for these connections.

Social media has been defined as a “collection of Web-based technologies that share a user-focused approach to design and functionality, where users can actively participate in content creation and editing through open collaboration between members of communities of practice” [4]. Social networking sites, such as Facebook and Twitter, blogs, wiki’s and many other interactive Web-based technologies are encompassed by this term. Social media is already well established as a powerful communication tool. In 2012, Twitter grew to over 200 million monthly users, and Facebook hosted over one billion users [5]. In contrast to journal articles, which predominantly facilitate top-down and one-way communication, social media may provide a forum for two-way discussion and feedback. Social media provides an avenue for information sharing that is not limited by geographical borders, potentially providing a convenient and cost-effective alternative to attending face-to-face conferences.

There has been a substantial growth in the use of social media within health care [5-7]. Research has demonstrated over 140 uses in health care for Twitter alone [6]. Provision of health education resources for patients and professionals, recruitment of patients to research studies, reporting of real-time flu trends, public outreach campaigns, and online consultations are only a few of the applications of social media in health care [6,7]. However, few studies have investigated attitudes and motives behind social media engagement in the health professions [5,8,9]. Most literature on the use of social media in health education has focused on undergraduate medical education [4], and although favorable results have been reported with regard to learner attitudes, knowledge, skills, and satisfaction [4,10], many of these studies lack methodological rigor [4]. No studies to date have investigated the use of social media in translating research evidence to clinical practice incorporating perspectives of health professionals from differing roles, disciplines, and nationalities.

Social media may assist in enhancing interaction and collaboration between health researchers and clinicians. This research aimed to explore health researchers’ and health professional clinicians’ current use of social media and their beliefs and attitudes towards the use of social media in professional contexts. We were particularly interested in exploring factors that might influence the use of social media and the future potential of social media to convey research evidence to those at the point of care. This would help us understand and subsequently utilize opportunities that social media may provide in improving the translation of research evidence to clinical practice.

## Methods

### Participants

Health practitioners (clinicians) who practice in the professional disciplines registered by the Australian Health Practitioner Regulation Agency (AHPRA) [11] were invited to take part in this study. Undergraduate students were eligible to participate if they were actively engaged in providing clinical care in a professional health care setting. Health researchers involved in formalized health care research were also invited to participate. While the invitation to participate was distributed in Australia, Malaysia, and India, participants from any geographical location were eligible to participate.

### Procedure

Ethical approval for the study was granted by the Monash University Human Ethics Committee (CF 14/1372 - 2014000640). The two phases of the data collection included an anonymous Web-based questionnaire and an interview. Since no existing validated survey was suitable for this study, an original questionnaire was developed by the researchers. The questionnaire consisted of 19 items with varying response types from which both quantitative and qualitative data were obtained (see Multimedia Appendix 1 for questions and response options). The questionnaire gathered demographic details on role, area of practice, age, gender, and country of residence. The questionnaire also gathered data on participants’ current social media use, and attitudes and beliefs regarding the use of social media in professional contexts, with an emphasis on using social media to communicate research evidence. At the close of the survey, participants were invited to provide contact details to participate in the interview phase of the data collection. Any contact details recorded were not linked to the previously recorded survey responses.

A link to the questionnaire was distributed by email. The email invited potential participants to take part in the questionnaire, as well as a link for those indicating that they were declining to participate. For those participants who chose to decline, an option was available for them to volunteer their reason for not participating.

The invitation to participate was distributed to research centers, clinical and educational affiliates, and departments of Monash University, Faculty of Medicine, Nursing and Health Sciences, Australia; Monash University Malaysia; and Swami Vivekanand National Institute of Rehabilitation Training and Research (SVNIRTAR), India. Health professional associations and peak bodies that represent professions registered with AHPRA were also contacted to distribute the invitation to participate.

As part of an action research cycle, results of preliminary data collection were used to develop semistructured interview questions for in-depth exploration of themes pertaining to professional development and professionalism arising from the questionnaire. The interview questions were an original script and are provided in Multimedia Appendix 2.

Participants from Australia, India, and Malaysia who consented to participate in an interview were stratified by country and role and selected at random. Individual interviews of approximately 20 minutes were conducted via telephone until saturation of themes occurred. Data collected in multiple countries allowed for validation of themes in international contexts. Data were audio recorded, transcribed, and de-identified prior to analysis.

The online questionnaires were open between June and November 2014. Interviews were conducted between July and October 2014.

Quantitative data were analysed using SAS statistical software [12]. The Cochran-Mantel-Haenszel (row mean scores) test was used to explore associations between demographic factors (country, age, gender, graduate status, and role) and the ordinal responses to questions. The corresponding *P* values were calculated using a chi-square distribution. Thematic analysis of qualitative data was conducted independently by 2 researchers who then discussed outcomes and arrived at a consensus regarding themes. Analysis was conducted according to the guidelines described by Braun and Clarke [13]. All representative quotes are reported verbatim to illustrate and provide context for derived themes.

## Results

### Overview

The invitation to participate was sent to 72 research centers, 65 heads of departments, 97 professional organizations, and 293 clinical or educational affiliates of Monash University Australia, Monash University Malaysia, and SVNIRTAR. Participants were able to select options within the emailed invitation to either accept or decline the invitation to participate. A total of 856 participants accepted the invitation to participate, which linked them through to the data collection survey; 125 potential participants elected to decline the invitation. Using the number of participants who accepted or declined the invitation to participate, the response rate for the survey was 87.3%. The reasons for declining to participate (participants could select more than one option) included lack of time (49/125, 39.2%), no interest in the study (40/125, 32.0%), felt the study was not relevant to them (31/125, 24.8%), and other reasons (15/125, 12.0%).

### Demographics

Over half of the participants were from Australia (542/856, 63.3%) and over half of all participants were clinicians (536/856, 62.6%). There was a high representation of females (522/856, 61.0%) and those aged 34 or younger (562/856, 65.7%). The demographic details of participants are listed in Table 1.

**Table 1:** Demographic details of participants.

		Australia, n (%)^a^	India, n (%)	Malaysia, n (%)	Other, n (%)	Total, n (%)
Total participants by country		542 (63.3)	166 (19.4)	90 (10.5)	58 (6.8)	856 (100)
**Role**						
	Clinician^b^	366 (42.8)	71 (8.3)	67 (7.8)	32 (3.7)	536 (62.6)
	Researcher	76 (8.9)	30 (3.5)	14 (1.6)	7 (0.8)	127 (14.8)
	Multiple^c^	76 (8.9)	61 (7.1)	4 (0.5)	13 (1.5)	154 (18.0)
	Not stated	24 (2.8)	4 (0.5)	5 (0.6)	6 (0.7)	39 (4.6)
**Student status**						
	Undergraduate	220 (25.7)	10 (1.2)	56 (6.5)	11 (1.3)	297 (34.7)
**Discipline**						
	Medicine	134 (15.7)	28 (3.3)	51 (6.0)	10 (1.2)	223 (26.1)
	Allied Health^d^	201 (23.5)	78 (9.1)	6 (0.7)	10 (1.2)	295 (34.5)
	Nursing	28 (3.3)	1 (0.1)	0 (0.0)	7 (0.8)	36 (4.2)
	Medical Research	54 (6.3)	4 (0.5)	8 (0.9)	4 (0.5)	70 (8.2)
	Other^e^	104 (12.1)	50 (5.8)	14 (1.6)	20 (2.3)	188 (22.0)
	Not stated	21 (2.5)	5 (0.6)	11 (1.3)	7 (0.8)	44 (5.1)
**Age**						
	≤24	208 (24.3)	16 (1.9)	53 (6.2)	9 (1.1)	286 (33.4)
	25-34	137 (16.0)	105 (12.3)	21 (2.5)	13 (1.5)	276 (32.2)
	35-44	88 (10.3)	34 (4.0)	10 (1.2)	13 (1.5)	145 (16.9)
	45-54	66 (7.7)	8 (0.9)	5 (0.6)	10 (1.2)	89 (10.4)
	55-64	34 (4.0)	3 (0.4)	1 (0.1)	12 (1.4)	50 (5.8)
	65+	8 (0.9)	0 (0.0)	0 (0.0)	0 (0.0)	8 (0.9)
	Not stated	1 (0.1)	0 (0.0)	0 (0.0)	1 (0.1)	2 (0.2)
**Gender**						
	Male	168 (19.6)	109 (12.7)	33 (3.9)	21(2.5)	331(38.7)
	Female	372 (43.5)	56 (6.5)	57 (6.7)	37 (4.3)	522 (61.0)
	Not stated	2 (0.2)	1 (0.1)	0 (0.0)	0 (0.0)	3 (0.4)

^a^All percentages are based on the total number of participants.

^b^The clinician category includes health practitioners in the professional disciplines registered by AHPRA and undergraduate students in those disciplines involved in clinical care.

^c^The multiple role category includes participants who identify as a clinician and researcher, or who have other roles in addition to clinician or researcher.

^d^The definition of Allied Health for this study is health care professions registered with AHPRA, excluding medicine and nursing.

^e^Includes responses where area of practice stated but discipline was unclear.

### Use of Social Media

**Figure 1:**
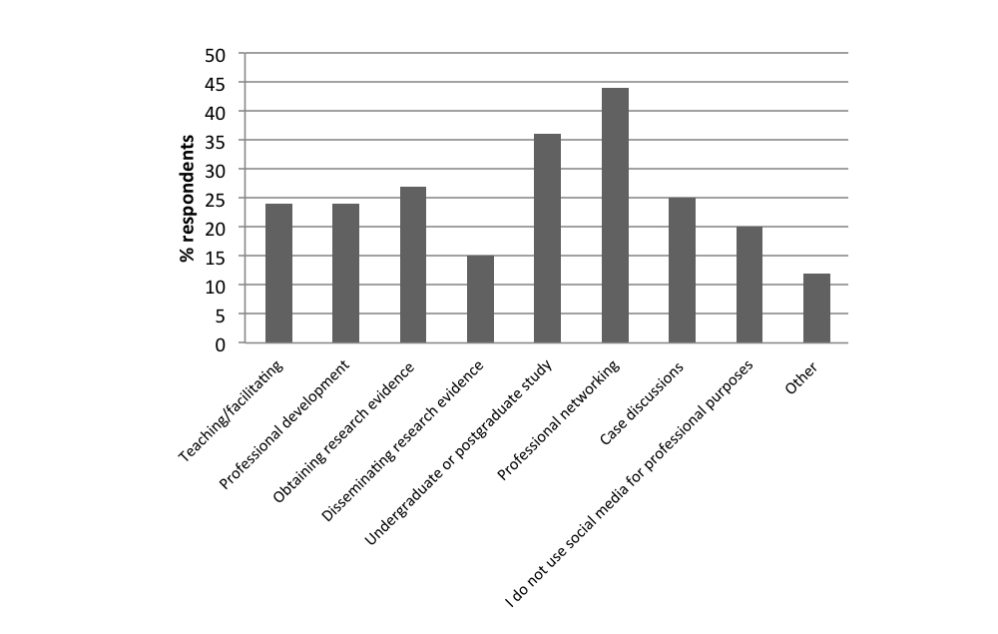
Categories of professional use of social media nominated by respondents.

Most respondents (749/840, 89.2%) reported using social media for recreational purposes, with 80.0% (682/852) of participants reporting use for one or more professional purposes. The most frequent use of social media in a professional context was for professional networking (44.1%, 376/852), followed by undergraduate or postgraduate study (306/852, 35.9%). Over a quarter (229/852, 26.9%) of participants used social media for obtaining research evidence, and 15.0% (128/852) of participants used social media for disseminating research evidence. Almost a quarter (201/852, 23.6%) of participants used social media for other professional development. The use of social media by respondents for professional purposes is shown in Figure 1.

Facebook was the social media platform most commonly used for both recreation (710/822, 86.4%) and professional purposes (363/779, 46.6%). YouTube was the second most used site for recreation (565/822, 68.7%) and professional purposes (359/779, 46.1%). When given a choice of platform for obtaining research information, Facebook was the most selected social media platform (227/848, 26.8%).

For respondents who accessed social media for recreation, the most frequent pattern of use, by 49.1% of participants (409/833), was to access social media more than once per day. For professional purposes the most frequent pattern of use, by 30.6% of participants (250/816), was to access social media a few times per week. A relationship between country and frequency of social media use for professional purposes was found. A total of 86.5% (77/89) of Malaysian and 76.7% (122/159) of Indian participants accessed social media a few times per week or more, compared to 56.1% (287/512) of Australian participants. A relationship between professional use and age, and professional use and graduate status was also found. Those in the <25 year age group were the most frequent users of social media for professional use, with 71.8% (199/277) using social media a few times a week or more. Professional usage frequency reduced with increasing age. More undergraduate students (221/291, 75.9%) used social media for professional purposes a few times per week or more compared to those who were not undergraduate students (292/525, 55.6%). While no difference was found between males and females for frequency of recreational use of social media, χ^2^_1_=1.38, *P*=.24), 66.0% (204/309) of males used social media for professional purposes a few times a week or more compared with 60.7% (306/504) of females (χ^2^_1_=4.57, *P*=.03). Professional usage patterns of social media were found to be unrelated to role (χ^2^_2_=2.76, *P*=.25). Table 2 shows the frequency of social media use in professional contexts by category.

For recreational purposes, most respondents read online material only (357/833, 42.9%) or contributed small amounts (356/833, 42.7%). For professional purposes, most participants also read online material only (445/843, 52.8%) or contributed small amounts (265/843, 31.4%). Participants aged <25 years contributed least to professional on line material, compared with participants in other age categories, with 95.2% (259/272) only reading or contributing small amounts to online material (χ^2^_4_=11.2, *P*=.02). Table 3 shows contributions to online material for professional purposes by age.

**Table 2:** Frequency of use of social media for professional purposes (the percentage shown is the percent of respondents for each row).

		Never, n (%)	Less than once month, n (%)	A few times per month, n (%)	A few times per week, n (%)	About once per day, n (%)	More once per day, n (%)	χ^2^	DF	*P*
**Country**										
	Australia	63 (12.3)	41 (8.0)	121 (23.6)	165 (32.2)	75 (14.7)	47 (9.2)	63.76	3	<.001
	India	8 (5.0)	4 (2.5)	25 (15.7)	47 (29.6)	39 (24.5)	36 (22.7)			
	Malaysia	4 (4.5)	1 (1.1)	7 (7.9)	28 (31.5)	26 (29.2)	23 (25.8)			
	Other	13 (24.1)	5 (9.3)	11 (20.4)	9 (16.7)	5 (9.3)	11 (20.4)			
**Age**										
	<25	18 (6.5)	9 (3.3)	51 (18.4)	96 (34.7)	64 (23.1)	39 (14.1)	20.1	4	<.001
	25-34	29 (11.2)	16 (6.2)	52 (20.2)	70 (27.1)	48 (18.6)	43 (16.7)			
	35-44	18 (13.0)	10 (7.2)	29 (20.9)	47 (33.8)	18 (13.0)	17 (12.2)			
	45-54	15 (17.2)	8 (9.2)	20 (23.0)	24 (27.6)	11 (12.6)	9 (10.3)			
	55+	8 (15.1)	8 (15.1)	11 (20.8)	12 (22.6)	4 (7.6)	10 (18.9)			
**Gender**										
	Male	33 (10.7)	11 (3.6)	61 (19.7)	90 (29.1)	60 (19.4)	54 (17.5)	4.57	1	.03
	Female	55 (10.9)	40 (7.9)	103 (20.4)	158 (31.4)	85 (16.9)	63 (12.5)			
**Graduate status**										
	Undergraduate	16 (5.5)	9 (3.1)	45 (15.5)	116 (39.9)	66 (22.7)	39 (13.4)	18.46	1	<.001
	Postgraduate / non student	72 (13.7)	42 (8.0)	119 (22.7)	134 (25.5)	79 (15.1)	79 (15.1)			

**Table 3:** Contribution to online material for professional purposes by age.

Age^a^	I read online material only			I contribute small^b^ amounts to online material			I contribute large amounts to online material		
	n	% age group^c^	% total participants	n	% age group	% total participants	n	% age group	% total participants
<25	173	63.6	20.2	86	31.6	10.0	13	4.8	1.5
25-34	132	54.3	15.4	84	34.6	9.8	27	11.1	3.2
35-44	70	56.0	8.2	45	36.0	5.3	10	8.0	1.2
45-54	39	48.8	4.6	32	40.0	3.7	9	11.3	1.1
55+	30	60.0	3.5	17	34.0	2.0	3	6.0	0.4

^a^n=2 did not provide age and are not included in the analysis.

^b^Participants were not given a specific definition of a “small amount” or “large amount”.

^c^Percent of age group that reported interacting with online material.

### Attitudes and Beliefs Toward Social Media for Professional Purposes

The majority (692/851, 81.3%) of respondents felt confident using social media for recreation compared with 58.2% (496/852) who felt confident using it for professional purposes. Confidence using social media for recreation and professional use reduced with increasing age (χ^2^_4_=134.5 and 31.7 respectively, *P*<.001 for both).

Just over half the participants (449/842, 53.3%) felt a need for further training to be able to use social media for professional development. Participants from all age categories expressed a need for training, but this was highest in the 45-54 years age category (60/83, 72.3%) and lowest in the <25 years age category (121/280, 43.2%; χ^2^_4_=34.1, *P*<.001).

Participants rated social media as the least useful method for staying up to date with research evidence (average rating 2.8 where 1 is not at all useful and 4 is very useful) compared with journals (3.6), mentors (3.4), conferences (3.3), and in-service programs (3.1).

Most respondents (729/843, 86.5%) felt the need to create connections between health researchers and clinicians. Almost all participants (810/845, 95.9%) also saw a role for social media in disseminating research evidence or obtaining clinical information; however, 14.5% (123/848) reported that they would not use social media for obtaining research or clinical information.

**Figure 2:**
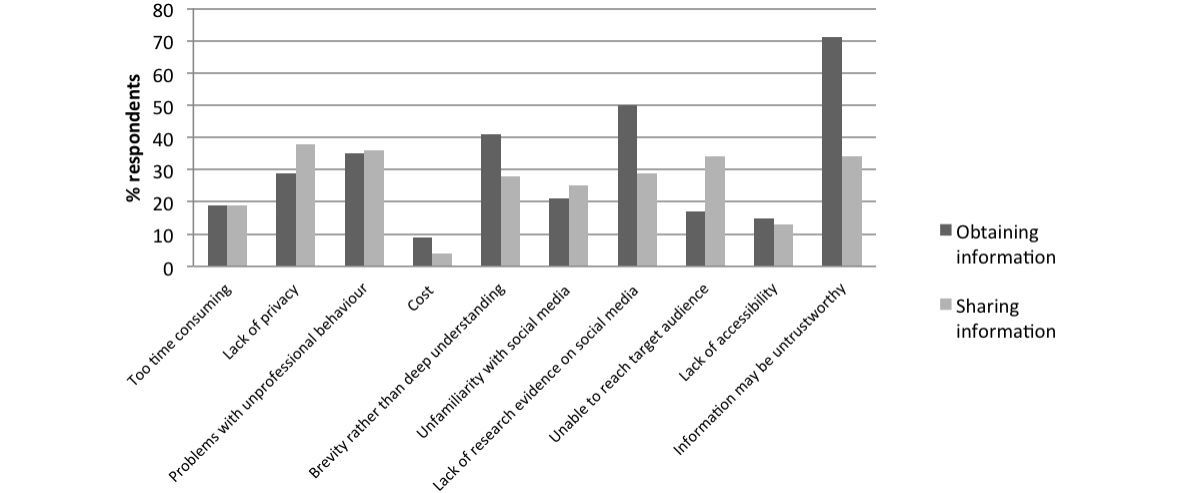
Obstacles to obtaining or sharing research or clinical information nominated by respondents.

The biggest obstacle to obtaining clinical or research information via social media was a felt to be information being untrustworthy (596/839, 71.0%), while the biggest obstacle to sharing clinical or research information via social media was a lack of privacy (305/807, 37.8%). Cost was identified as being the smallest barrier. Barriers to using social media for sharing or obtaining clinical or research information are described in Figure 2.

Most participants considered professionalism in their professional social media use. Most respondents (569/842, 67.6%) were concerned about how material they contribute to social media would represent them. Just over half of respondents (463/841, 55.1%) believed that the material they contributed to social media may positively influence their career and 45.5% (381/838) felt it may have a negative effect.

### Thematic Analysis

#### Overview

Interviews were conducted with 27 participants from Australia (9 researchers, 18 clinicians, including 10 students), 28 participants from India (5 researchers, 20 clinicians, including 10 students and 3 “other” roles), and 14 participants from Malaysia (14 clinicians, including 10 students). A saturation of themes was obtained within the clinician, student, and researcher subcategories, and between countries.

Thematic analysis of interviews from Australia, India, and Malaysia revealed three major themes. The themes were consistent between countries and roles. Quotes have been provided to illustrate each theme and are coded by country (A=Australia, I=India, M=Malaysia), role (C=clinician, S=student, R=researcher), and a participant number.

#### Profile of Social Media

Participants felt that the use of social media in health professions is still developing, particularly with regards to health professionals’ understanding of how to use social media in professional contexts, ensuring professional conduct when using social media, and the regulation of professional social media environments: “I think there’s a fledgling growth in using social media for some professional stuff but I think it’s still in its infancy” (AC2) and “I think it will continue to grow and become a more—I think people will begin to view it as a more reputable source” (AS5).

Participants also felt that there was a stigma attached to using social media for professional use. They felt that using social media was seen as being unprofessional and that information obtained via social media was less valid than that from other sources such as peer-reviewed journals. This perception was held by participants from Australia, India, and Malaysia, and by students, clinicians, and researchers. Some participants also felt that it was a source of information that would be utilized by “younger” health researchers and clinicians only: “I don’t think it’s professional at all” (MS2) and “This is a generational thing…you can’t teach an old dog a new trick” (IC1).

#### Contributions, Concerns, and Considerations

Participants felt that social media held value for health researchers and clinicians in professional contexts. Features such as accessibility and convenience, the ability to disseminate information quickly to a large audience, and the opportunity to develop networks were key benefits of social media: “One of the real values that I think that social media can offer is that trans-disciplinary and multi-disciplinary conversations in research” (AS10).

Participants were wary of the trustworthiness of the content of social media posts such as research reports due to its lack of regulation. They felt brevity of messages was a concern in establishing validity of information and that some information may be anecdotal or used to support certain agendas: “if you see something posted or shared or Tweeted or whatever...that doesn’t mean it is the best evidence on something…You have to realize who is posting it, what potential agendas may be, or where that article or topic or information might fit within your field of practice” (AR5).

Respondents also reported apprehension over the mixing of personal and professional lives and the need to use social media in a manner that would allow distinction between the two. Participants were also concerned about their digital identity, including being misunderstood or misinterpreted, and being held accountable for their social media posts: “I think there’s always the worry that the lines might blur between professional and social. It only takes one mistake, one photo, one silly comment, and it can lead to a whole lot of other problems and outcry” (AS3).

Participants felt that social media could not replace face-to-face interactions. More than half (40/69, 58.0%) of participants interviewed would prefer to attend a conference in person rather than via social media. Reasons for this choice included the potential to build relationships with colleagues resulting from rich interactions that may arise from face-to-face exchanges. However, it was acknowledged that social media may be a more convenient and less expensive alternative to attending conferences face to face. Participants also felt that using social media to broadcast conference proceedings may also increase audience size and dissemination of information: “[social media is] more convenient and there’s no extra expenses in terms of travelling” (MS6).

Many participants felt they had not been “trained” to use social media for professional purposes. This included the choice of social media platforms to use and how to use them in professional contexts. If training in the professional use of social media were to occur, participants felt that this training could either be face to face or online.

I don’t think we’ve been really trained to use social media to look up articles or to look up research…So we just kind of fiddle around and we jump on things and we try out different things and we don’t know exactly if we’re using it to the full capacity…So there’s definitely a huge need for more training in this area

AC5

#### Best Practice for Professional Use of Social Media

Participants felt that social media could and should be used in professional contexts if there were specific platforms for professional use, run by accredited or respected bodies or peers. These platforms would be standardized, content controlled, restricted access, peer reviewed, and contain links to full sources of information: “Maybe it would be interesting to have some platforms from different institutions, like universities…That would also make trustworthiness increase” (AR6) and “there’d need to be really clear links, I think direct links, to the actual source of the evidence so that that could then be accessed directly” (AC3).

## Discussion

### Principal Findings

The results of this study demonstrate a high level of engagement by health researchers and clinicians with social media in both recreational (89.2%) and professional (80.0%) contexts. However, far fewer use social media for obtaining or distributing research evidence, and there are different patterns of use by country, age, gender, and graduate status. A key barrier in using research evidence obtained via social media may be the perceived untrustworthiness of information obtained via this medium.

Wide variation in rates of social media use are reported in existing literature with students showing higher use (64-97%) [14,15] than clinicians (13-47%) [14] and researchers (51.9%) [8]. This study also found a high level of professional social media use by undergraduate students and those in the <25 year age category. This result may be reflective of an increasing use of social media within health care education courses [4]. Although social media has been considered to be a “Generation Y” phenomenon [16], a need for training in the professional use of social media was expressed by participants of all ages, not just older subgroups. Few training programs in social media exist, but those that do, such as the “Friending Facebook” course at Penn State Hershey Medical Center have achieved favorable results [17].

Professional use was most frequent by Malaysian and Indian participants. This is reflective of worldwide trends of social media use, which demonstrate that the average number of daily hours spent on social media is highest in Malaysian residents, followed by Indian and Australian residents [18]. The higher proportion of participants in the 34 and under age category from Malaysia and India may also have influenced this result. Males were more frequent users of social media for professional purposes than females, despite similar rates of use for recreation. The high proportion of male compared with female participants from India, where professional use rates are high, may have influenced this result.

The results of this study demonstrate that networking was the predominant motive for social media use, a result comparable to previous studies [5,9]. Most health researchers and clinicians who participated in this study (86.5%) consider it important to create professional connections, which also correlates with this result. While networking may be useful in building a health professional’s profile or building relationships, social interaction via these networks may also impact upon the translation of evidence to clinical practice. Social influence is a powerful change inhibitor or facilitator [19] and the opinions of peers and leaders play a major part in influencing individual practitioners’ behavior, especially with regards to acting on new information [19].

Fifteen percent of health researchers and clinicians in this study indicated that they currently distribute research findings, while 26.9% obtain research evidence via social media. The rationale for the limited use of social media in conveying research evidence may lie within the perceived barriers to its use. Trustworthiness of information obtained via social media was a key concern for participants in this study. The open access environments of social media allow the circulation of both evidence-informed and opinion-informed messages. The quality and validity of Web-based health information has been of concern since the Internet became publicly accessible in the mid-1990s [6].While these concerns may be valid, obtaining evidence-based information from traditional sources such as academic journals does not necessarily guarantee its quality. “Predatory journals” may publish articles of poor quality in return for payment [20]. Clinicians must possess both the time and skills to decipher what is both valid and reliable, regardless of source. These barriers to social media use have been identified in previous research [5]. Participants in this study had specific ideals regarding best practice for the use of social media in disseminating and obtaining research evidence in future. These included particular standards such as content-controlled sites run by accredited or respected bodies. Those seeking to disseminate research evidence should consider these ideals in the development of social media platforms and content, and application of these standards may assist in reducing the “unprofessional” stigma of these media. Initiatives to improve the quality of Web-based information exist, such as validated information sites, for example Medpedia (a Harvard, Stanford, University of Michigan, and UC Berkley initiative) [6]. The World Health Organization has also proposed to instigate a regulated health domain for validated health information [6]. However, health professionals must be aware of these sites, and restricting user-generated content may limit peer interaction. As the uptake of evidence-based information is more likely from a participatory educational program [19], increased regulation may limit the educational value of these sites.

Despite the perceived barriers to using social media for communicating research or clinical information, most participants in this study saw a role for social media in obtaining or disseminating research evidence, which is considerably more than reported in previous literature [8]. Social media has several features that enhance its utility for dissemination of research evidence, which may have contributed to this result. Cost was identified as the smallest barrier to professional use. The cost of scholarly journals can be high and continue to increase in price [21]. A World Health Organization investigation of the lowest income countries reported that 56% of institutions had no subscription to international journals, and 21% had an average of only two subscriptions [22]. Social media may be a cost-effective alternative to journal subscription, as access to social media sites are free (given an Internet connection and Web-enabled device). This may enable greater equity in distribution of health information globally.

Participants also identified accessibility and the rapid dissemination of information as benefits of social media. Social media is available to anyone with an Internet connection and Web-enabled device, a feature of particular benefit to health professionals in geographically isolated areas. Online communities of practice have proven to enhance the use of EBP, which may be used within rural and remote areas [23], although paradoxically, these may be areas where technological infrastructure does not support fast or reliable Internet connections. Social media also distributes information rapidly. With a median time from study completion to journal publication of 2.4 years [24], social media may have the potential to reduce the time from knowledge creation to implementation in clinical practice, compared with traditional methods of evidence-based information dissemination.

### Limitations

This study has several limitations. Although participation was open to participants from all geographical locations, the invitation to participate was distributed in Australia, India, and Malaysia, resulting in data predominantly from participants in these regions. Future studies should validate this study’s findings with other populations. In addition to this, participant discipline could not always be ascertained, therefore the data may include responses from participants in professions other than those registered with AHPRA. The questionnaire was presented online, therefore participants with reduced information technology access or skill may have been unable to participate. This may have resulted in selection bias towards those who favor the use of social media. While an effort was made to calculate a response rate based on the two questionnaires used, not all non-responders will have been captured. Therefore, the response rate may be lower than that reported. Two research assistants conducted the interviews with participants in Australia; however, use of local interviewers in India and Malaysia may have affected the information generated from interview data. Poor phone connections in some cases may have led to misinterpretation of participant intention. A further limitation to this study is that the analysis of findings has remained broad, as many themes arising from the data were consistent between subgroups of participants. However, this limits the depth of understanding of the findings in relation to these subgroups. Future studies may include geographical or role-based analysis of findings on this topic to contribute to existing literature.

### Conclusions

With an average of 17 years required to incorporate 14% of research findings into clinical practice [3], it is evident that there is a disparity between health care knowledge and health care practice. Social media may assist in “filling the gaps” left by traditional methods of research dissemination, by providing a rapid, accessible, cost-effective medium with which to disseminate information. Social media for knowledge translation may also provide an avenue for discussion, collaboration, and peer-review that may enhance learning and acceptance of new information.

This study found that a large majority of health researchers and clinicians use social media in recreational and professional contexts. This study has also found relationships between age, gender, country of residence, and graduate status with use of social media in professional contexts. Younger age, male gender, undergraduate status, and residency in Malaysia or India were indicators of high use of social media for professional purposes. However, this finding should not limit the investigation or use of social media in communicating research information to these subgroups. This study has also demonstrated that a vast majority of health researchers and clinicians feel that social media has a role to play in the communication of research evidence, but they lack trust in the reliability and validity of information on social media. This is a valid concern and may limit the use of social media in translating research evidence to clinical practice. Therefore, methods for improving the “reputation” of social media for professional use should be investigated. This study has found that these methods may include tailoring of social media platforms and content to enhance the utility for professional purposes and provide clinicians and researchers with greater trust and safety in use. Training programs may also assist in increasing the number of health professionals using social media for obtaining and communicating research evidence. Future research should also investigate the efficacy of social media in communicating research evidence and the impact on clinician’s attitudes, knowledge, and clinical practice.

## Multimedia Appendix 1

Study questionnaire.

## Multimedia Appendix 2

Semi-structured interview questions.

## Abbreviations

AHPRA: Australian Health Practitioner Regulation Agency
EBP: evidence-based practice
SVNIRTAR: Swami Vivekanand National Institute of Rehabilitation Training and Research
