# Supplementary material for: The Acceptability Among Health Researchers and Clinicians of Social Media to Translate Research Evidence to Clinical Practice: Mixed-Methods Survey and Interview Study
Source: J Med Internet Res. 2015 May 20;17(5):e119. doi: 10.2196/jmir.4347 (PMC4468567; doi:10.2196/jmir.4347)
Supplement: Supplementary file 1 [file jmir_v17i5e119_app1.pdf]

Multimedia appendix 1. Survey questions and responses

Question	Response options
Which of the following best describes your current occupational and / or academic status? (you may select more than one option)	Undergraduate<br>Postgraduate clinical trainee<br>Postgraduate research trainee<br>Clinician<br>Researcher<br>Educator<br>Other (please specify)
Please describe your principal area of clinical practice, research or study (e.g. nursing, sport and exercise medicine, cancer research)	Open text response
What is your age?	Under 18<br>18 – 24<br>25 – 34<br>35 – 44<br>45 - 54<br>55 – 64<br>65 – 74<br>75 or older
What is your gender?	Female<br>Male
In what country / region do you predominantly reside?	Australia<br>India<br>Malaysia<br>UK<br>Other (please specify)
Do you use social media for recreation?	Yes<br>No
Please indicate for which professional purposes you use social media (you may select more than one)	Teaching / facilitating<br>Professional development (please provide further details in comments section)<br>Obtaining research evidence<br>Disseminating research evidence<br>Undergraduate or postgraduate study<br>Professional networking<br>Case discussions<br>I do not use social media for professional purposes<br>Other (please specify)
For the following purposes, which of the following social media tools / networks do you use? (you may select more than one)<br>• Recreation<br>• Professional purposes	Facebook<br>Twitter<br>Google+<br>You Tube<br>Blogs<br>Podcast<br>LinkedIn<br>N/A<br>Other (please specify)
For the following purposes, how often do you use social media?<br>• Recreation<br>• Professional purposes	Never<br>Less than once per month<br>A few times per month<br>A few times per week<br>About once per day<br>More than once per day<br><br>Comments
Please indicate whether you agree or disagree to the following statements. Please feel free to comment on your answers.<br>• I feel confident using social media for recreation<br>• I feel confident using social media for professional purposes	Strongly disagree<br>Disagree<br>Neither disagree nor agree<br>Agree<br>Strongly agree<br>N/A

• I would require further training to use social media for my own professional development	Comments
For the following purposes, please indicate your level of contribution to on-line material<br><br>• Recreation<br>• Professional purposes	I read on-line material only<br>I contribute small amounts to on-line material<br>I contribute large amounts to on-line material<br>N / A<br><br>Comments
Please select the usefulness of the following methods you use to stay up to date with research evidence<br><br>• Conferences<br>• Inservice programs<br>• Mentors<br>• Journals<br>• Social Media	Not at all useful<br>Not very useful<br>Somewhat useful<br>Very useful<br><br>Comments
Do you feel that social media has a role in the dissemination of research evidence or obtaining clinical information? Please comment on your answer.	No role at all<br>Minor role<br>Moderate role<br>Major role<br><br>Comments
If you could use only one of the following social media networks for obtaining research or clinical information, which would you use?	I would not use social media for obtaining research or clinical information<br>Facebook<br>Google+<br>Twitter<br>YouTube<br>Pinterest<br>Blogs<br>Podcast<br>Videochat<br>Other (please specify names of social media you would use, e.g. ResearchGate etc.)
Please indicate whether you agree or disagree with the following statements. Please feel free to comment on your answers.<br><br>• I am concerned about how material I contribute to social media represents me<br>• Material I contribute to social media may positively influence my career<br>• Material I contribute to social media may negatively influence my career	Strongly disagree<br>Disagree<br>Neither disagree not agree<br>Agree<br>Strongly agree<br><br>Comments
What are the main obstacles in using social media to obtain or share clinical or research knowledge? (you may select more than one answer)<br><br>• Obtaining clinical or research information<br>• Sharing research or clinical information	Too time consuming<br>Lack of Privacy<br>Problems with unprofessional behaviour<br>Cost<br>Brevity rather than deep understanding<br>Unfamiliarity with social media<br>Lack of research evidence on social media<br>Unable to reach target audience<br>Lack of accessibility<br>Information may be untrustworthy<br><br>Comments
As a current or prospective researcher, academic or clinician, how important is it for you to create connections with counterparts in areas outside your own (e.g. health researcher with clinicians)?	Very unimportant<br>Unimportant<br>Neither unimportant nor important<br>Important<br>Very important<br><br>Comments

Where did you find out about this survey? (you may select more than one answer)	Through my workplace<br>Through my place of study<br>From my professional association / society<br>Via social media<br>Other (please specify)
Do you have any other comments, questions, or concerns about social media raised by this survey that you're keen to share?	Open text response
