# Supplementary material for: The Acceptability Among Health Researchers and Clinicians of Social Media to Translate Research Evidence to Clinical Practice: Mixed-Methods Survey and Interview Study
Source: J Med Internet Res. 2015 May 20;17(5):e119. doi: 10.2196/jmir.4347 (PMC4468567; doi:10.2196/jmir.4347)
Supplement: Supplementary file 2 [file jmir_v17i5e119_app2.pdf]

Multimedia Appendix 2. Semistructured interview questions

1. "Firstly, can I confirm whether you are a clinician, researcher or student?"

2. "Our survey revealed that clinicians and researchers are more confident in using social media for recreation, than they are for professional purposes. Why do you think this is?

3. "What do you think it means to be professional when using social media?"

   If asked what the word professionalism means the following definition is given *"Professionalism means clinical or scientific excellence in any interaction with others."* If only negative aspects are mentioned, a supplementary question should be *"Can social media confer any positive aspects with regards to professionalism?"*

4. "People have indicated that they may like training in how to use social media for professional purposes. If you were to undertake training in how to use social media, in what format/medium do you think this should take and why?"

5. "Survey participants indicated that one of the current barriers to using social media to stay up to date with evidence was the trustworthiness of the information. What do you think would make the information more trustworthy and why? "

   If not mentioned then a supplementary question should be *"How would you assess the trustworthiness of professional information on social media?"*

6. "Survey participants indicated that they found using social media of almost equal usefulness as conferences for staying up to date with emerging evidence. If there were a conference in your area of expertise, that was going to be delivered via social media, would you prefer to go to the conference, or participate via social media? Why? "

7. "If you were a clinician or researcher with unique expertise in the field of the conference, would you consider being co-presenter to a social media audience? Why?"

8. "Do you have any other comments or ideas regarding using social media to translate research evidence to clinical practice that you would like to share?"
